# Facile Mesoporous Hollow Silica Synthesis for Formaldehyde Adsorption

**DOI:** 10.3390/ijms24044208

**Published:** 2023-02-20

**Authors:** Misun Kang, Jong-tak Lee, Jae Young Bae

**Affiliations:** Department of Chemistry, Keimyung University, Daegu 42601, Republic of Korea

**Keywords:** mesoporous silica, mesoporous hollow silica, room-temperature synthesis, formaldehyde adsorption

## Abstract

Formaldehyde emitted from household products is classified as a hazardous substance that can adversely affect human health. Recently, various studies related to adsorption materials for reducing formaldehyde have been widely reported. In this study, mesoporous and mesoporous hollow silicas with amine functional groups introduced were utilized as adsorption materials for formaldehyde. Formaldehyde adsorption characteristics of mesoporous and mesoporous hollow silicas having well-developed pores were compared based on their synthesis methods—with or without a calcination process. Mesoporous hollow silica synthesized through a non-calcination process had the best formaldehyde adsorption characteristics, followed by mesoporous hollow silica synthesized through a calcination process and mesoporous silica. This is because a hollow structure has better adsorption properties than mesoporous silica due to large internal pores. The specific surface area of mesoporous hollow silica synthesized without a calcination process was also higher than that synthesized with a calcination process, leading to a better adsorption performance. This research suggests a facile synthetic method of mesoporous hollow silica and confirms its noticeable potential as a support for the adsorption of harmful gases.

## 1. Introduction

Formaldehyde is broadly applied in wood processing, pharmaceuticals, cosmetics, clothing, and food [1,2,3]. However, in 2004, formaldehyde was classified as a potential carcinogen and teratogen by the World Health Organization (WHO). In particular, formaldehyde emitted from wooden or leather furniture in an indoor environment can have serious adverse effects on human health [4,5,6]. The International Agency for Research on Cancer (IARC) under the WHO has classified formaldehyde as a class one carcinogen that can cause fatal damage to organs such as the liver, kidney, eyes, nose, skin, and throat. Since 2015, Europe has banned the usage of formaldehyde [7]. Formaldehyde is emitted not only indoors but also outdoors, where the use of building materials for the construction of infrastructures such as roads and buildings is increasing [8,9]. However, as there is still no alternative material to formaldehyde, studies are being actively conducted to control the release of formaldehyde and to capture it to secure human safety.

Various studies are being carried out using nanomaterial supports to adsorb formaldehyde. Carbon nanomaterials such as carbon nanotubes [10,11,12] and graphene [13,14,15] have been recently employed in various attempts to adsorb formaldehyde. Zeolite [16,17,18], active carbon [19,20], and porous silica nanomaterials [21,22,23] are also utilized as support materials for adsorption. Formaldehyde with a boiling point of −19.5 °C has a gaseous state at room temperature. Thus, it should be collected through vapor adsorption. However, since moisture and formaldehyde compete for adsorption to adsorbers in a humid environment, amine functional groups are introduced on the adsorption support surface [24,25,26]. These amine functional groups can induce formaldehyde into imine bonds, which would be advantageous for chemical adsorption. The aforementioned carbon nanomaterials and various nanostructured materials are usually synthesized through costly and complicated processes, making them hard to be applied in real life. Conversely, mesoporous nanomaterials with a well-developed porous structure can adsorb a great amount of gas due to their large surface area. In particular, mesoporous silica nanomaterials have favorable properties such as an easy fabrication method and low-cost materials [27]. The porous silica nanomaterials, which we have reported previously [28], can be prepared by a simple method even at room temperature. They have large pore volumes that provide the potential to attach large numbers of amine functional groups. Since hollow silica nanomaterials possess large pores even internally, they are in the spotlight as advantageous materials for gas adsorption. Indeed, mesoporous hollow silica nanomaterials have been studied as carbon dioxide adsorption supports [29,30]. However, they face a lot of difficulties in practical use because tricky synthesis methods are needed for the unique structure of hollow silica nanomaterials.

In this study, mesoporous silica nanomaterials and mesoporous hollow silica nanomaterials were synthesized with an easy method. The surface properties of such silica nanomaterials with and without calcination were then compared. In addition, the potential of these mesoporous hollow silica nanomaterials as supports for formaldehyde adsorption supports was investigated.

## 2. Results and Discussion

**Figure 1:**
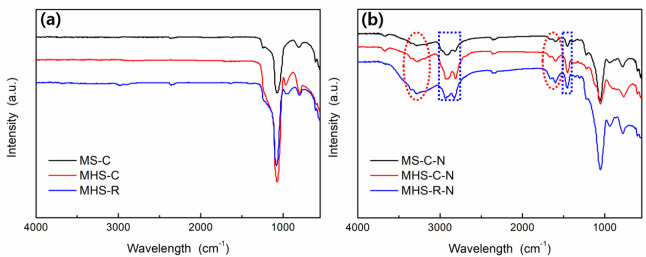
FT-IR spectra of mesoporous silica and mesoporous hollow silica: (**a**) Before amine functional group attachment; and (**b**) After amine functional group attachment.

Figure 1 shows Fourier transform infrared spectrometer (FT-IR) spectra of the synthesized mesoporous silica and mesoporous hollow silica samples with and without amine functional groups attached. The FT-IR spectra of mesoporous silica and mesoporous hollow silica before attaching an amine group are shown in Figure 1a. Peaks at 3000 to 2800 cm^−1^ representing C-H symmetric and asymmetric stretching vibrations, are not visible, confirming that the surfactant and residual organics were removed [31]. Peaks at 1070 and 810 cm^−1^ indicated symmetric and asymmetric stretching vibration of Si-O-Si, as shown in Figure 1b. The FT-IR spectra of mesoporous silica and mesoporous hollow silica after attaching amine functional groups showed C-H stretching and bending vibration peaks of the amine group at 2930, 2820, and 1450 cm^−1^ with N-H asymmetric (highlighted by red ovals) and symmetric stretching vibrations of the amine group at 1600, 1670 and 3100 to 3400 cm^−1^ (highlighted by blue rectangles) [32]. The spectra confirmed that the process of attaching the amine functional group was successfully carried out. Compared to the intensity of the peaks of MS-C-N, the peak intensity of MHS-C-N or MHS-R-N was stronger, meaning that more amine groups were attached to the hollow silica materials.

**Figure 2:**
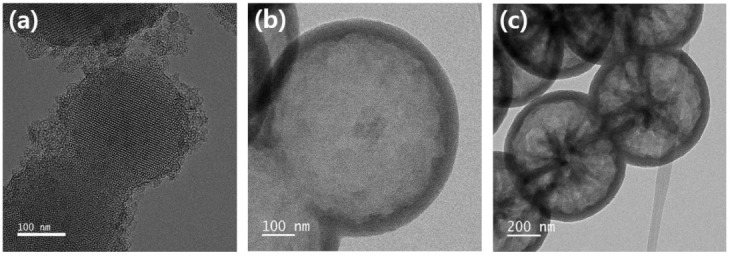
TEM images of mesoporous silica and mesoporous hollow silica: (**a**) MS-C, (**b**) MHS-C, and (**c**) MHS-R.

TEM images of mesoporous and mesoporous hollow silica nanomaterials are shown in Figure 2. The mesoporous silica materials had hexagonal lattices, as shown in Figure 2a, because the MS-C synthesized by the calcination process had regular pores of uniform size. As shown in Figure 2b,c, the hollows of MHS-C and MHS-R were approximately 630 nm in size and the shell thickness of both silica materials was also similar, ranging from 40 to 50 nm. Forming pores or hollows by surfactant-removal either by calcination using high-temperature heat or extraction using HCl/EtOH had no significant effect on the size of the hollows or the thickness of the shell. In magnified images, the MHS-C showed enlargement because of the calcination process, which gave sufficient thermal energy to build strong bonds. However, the siloxane bonds of MHS-R were not strong enough to withstand the energy of the electron beam. Thus, the lattice structure collapsed due to the magnified TEM image measurement. Despite the restricted information from TEM, it could be seen that MHS-R had an irregular pore structure compared to MHS-C.

**Figure 3:**
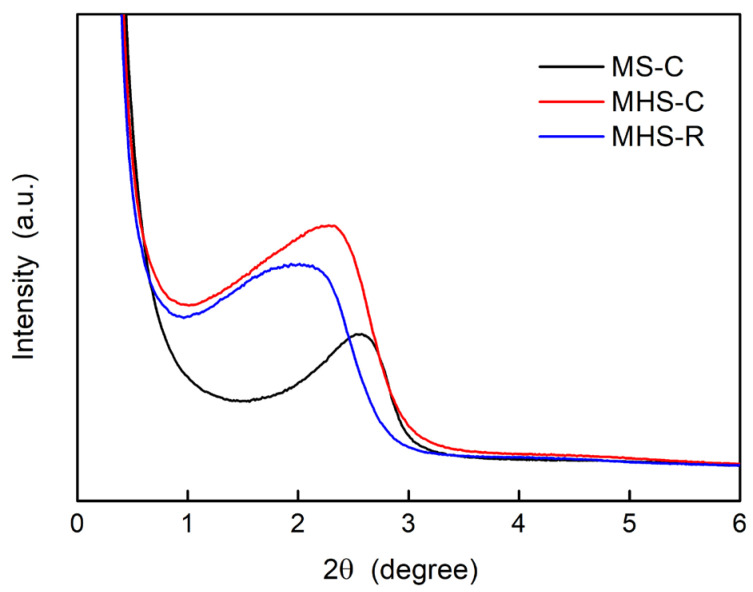
Low angle XRD patterns of mesoporous silica and mesoporous hollow silica nanomaterials.

Figure 3 shows low-angle XRD patterns confirming the presence of pores in all silica materials. Peaks for MS-C (black line), MHS-C (red line), and MHS-R (blue line) are shown at 2.5, 2.3, and 2.03, respectively. This result means that MS-C has the smallest pore size, MHS-C has an intermediate size, and MHS-R has the largest pore size. The sharper peak of MS-C indicates a uniform and small pore size, whereas the broader peak of MHS-R indicates varied and random sizes of pores. These XRD patterns are consistent with the TEM analysis results showing that MHS-R consists of random-sized pores including areas of collapsed pores. This was because MHS-R, synthesized without a calcination process, did not receive any energy to firmly stack Si-O-Si bonds or order these pores. Compared to the peaks of the MS nanomaterials, the peaks of the MHS nanomaterials showed a left shift, meaning large pores [28]. Although the same surfactant was used in the synthesis of these three silica nanomaterials, the hollow structures of the MHS were recognized as broader pores in the XRD patterns.

**Figure 4:**
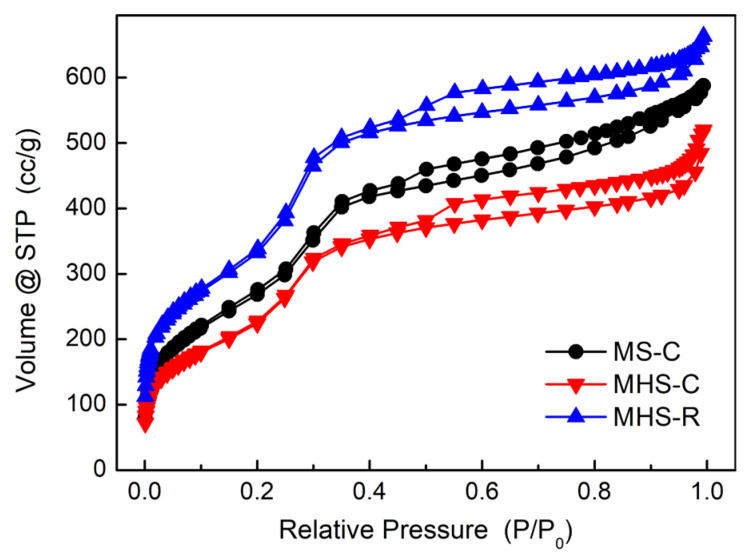
N_2_-adsorption-desorption isotherm graphs of mesoporous silica and mesoporous hollow silica materials.

N_2_-adsorption-desorption isotherm results of mesoporous silica and mesoporous hollow silica materials are depicted in Figure 4. The N_2_-adsorption-desorption isotherm results of the three silica nanomaterials were type IV with hysteresis loops. Comparing the area with relative pressures of 0 to 0.3, the order of the specific surface area was: MHS-R > MS-C > MHS-C. The separation of adsorption-desorption curves of the three silica nanomaterials appeared at the relative pressure point of 0.5, implying that all three silica materials consisted of mesopores of almost the same size. This result was different from that of the low-angle XRD analysis. This might be because the hollow volume inside MHS materials could not be analyzed with the N_2_ sorption method as the multiple outer layers with pores captured liquid nitrogen with low entropy [33]. Therefore, the N_2_ sorption results were caused only by pores of the outer multi-shell of the MHS materials.

**Figure 5:**
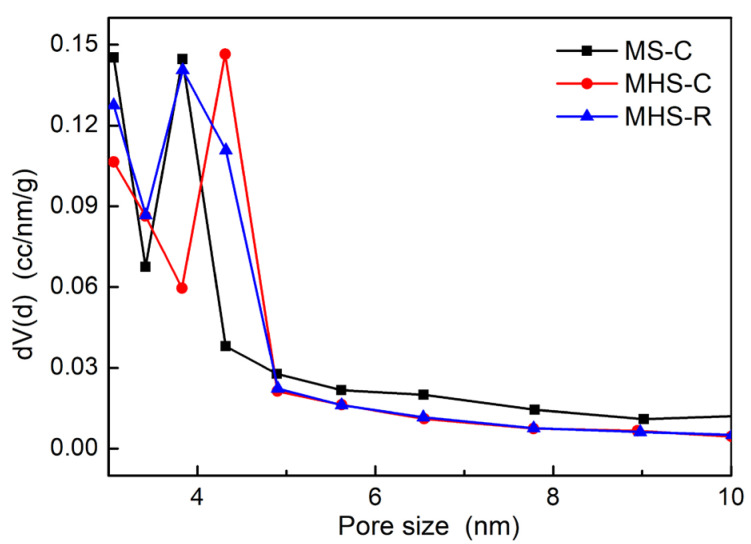
Pore size distributions of mesoporous silica and mesoporous hollow silica.

Pore size distributions were determined from the desorption graphs of the N_2_-adsorption-desorption isotherm shown in Figure 5. The specific surface areas, pore sizes, and pore volumes of the three silica nanomaterials are summarized in Table 1. Consistent with the previous results, MHS-R had the largest BET-specific surface area and MHS-C had the smallest BET-specific surface area. Since the calcination process leading to the growth of the silica nanoparticles reduced the specific surface area, MHS-R, which was synthesized with extraction and surfactant removal process at room temperature without a calcination process, could not grow nanoparticles. Therefore, MHS-R had the largest specific surface area, larger than MHS-C and MS-C. However, pore volumes caused by the same surfactant during the synthesis were mostly similar because the determined pore volumes of the MHS silica materials results accounted for only the outer multi-shell excluding the hollow interior volume.

**Figure 6:**
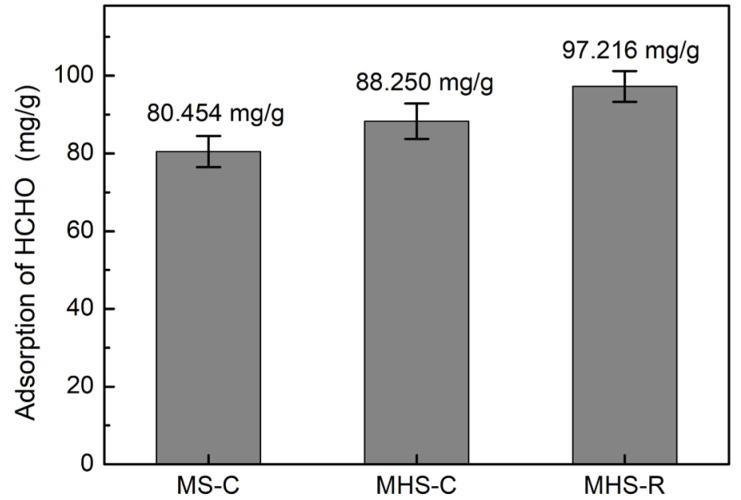
Formaldehyde adsorption results for mesoporous silica and mesoporous hollow silica materials.

Figure 6 depicts the adsorption results of formaldehyde gas in the order of MHS-R, MHS-C, and MS-C. Since mesoporous hollow silica materials had more internal large pores, they could be more advantageous for gas adsorption [34]. Internal large pores of MHS materials were observed in the TEM images and low-angle XRD. However, they could not be captured in the N_2_-sorption results. In the TEM images and low-angle XRD results, the pores of the mesoporous hollow silica materials were well established compared to those of the mesoporous silica materials. When comparing mesoporous hollow silicas, the adsorption performance is generally determined by the pore volume. However, since the two hollow silica materials had similar internal pore radii and shell thicknesses as determined from the TEM images, and, therefore, similar pore volumes, although only outer shell pores were involved, the specific surface area was the key to enhancing the adsorption of formaldehyde. The specific surface area of MHS-R was larger than that of MHS-C. In the FT-IR results shown in Figure 1, MHS-R had a stronger amine peak intensity than MHS-C. Therefore, the introduction of an amine functional group into MHS-R was more advantageous. The sufficient amine functional group content caused a large amount of adsorption of formaldehyde gas as shown in Figure 6 [25].

**Figure 7:**
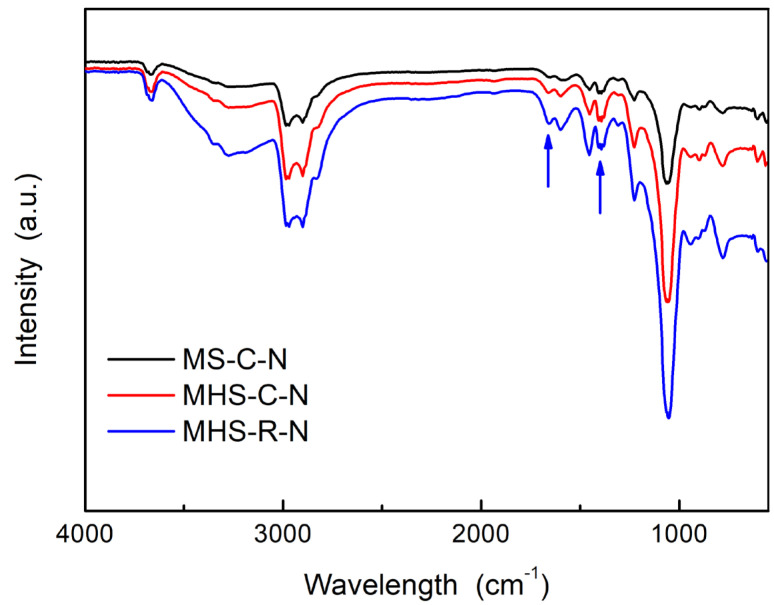
FT-IR spectra of mesoporous silica and mesoporous hollow silica nanomaterials after formaldehyde adsorption.

Figure 7 shows the FT-IR spectra of the mesoporous silica and mesoporous hollow silicas after formaldehyde adsorption. Compared to the FT-IR results of the mesoporous silica and mesoporous hollow silica nanomaterials in Figure 1b, the intensities of the peaks at 1663 cm^−1^ increased (indicated by the arrow on the left) due to the imine stretching as the product of the reaction between amine and formaldehyde [25,35]. This indicates that the MHS with amine functional groups reacted chemically with the adsorbed formaldehyde. Conversely, if the adsorption had occurred physically, there should be a peak in the region of 1725–1705 cm^−1^ representing a carbonyl bond. However, there are no peaks in this region. In addition, new peaks at 1399 cm^−1,^ new to the spectra shown in Figure 7 (indicated by the arrow on the right), represent the C-H stretching of aldehyde [36]. The FT-IR results after formaldehyde adsorption confirmed that the amine functional groups added into the silica nanomaterials adsorbed formaldehyde gas.

## 3. Materials and Methods

### 3.1. Materials

For the synthesis of hollow mesoporous silica, TEOS (tetraethyl orthosilicate, 98 wt%, Sigma-Aldrich-Korea, Seoul, Republic of Korea) and sodium silicate solution (28–30 wt%, DAEJUNG, Siheung-si, Republic of Korea) were used as silica precursors. To implement a hollow and internal pore structure, cetyltrimethylammonium chloride (CTACl, 25 wt%, Sigma-Aldrich-Korea, Seoul, Republic of Korea) was used as a surfactant. Hydrochloric acid (HCl, 35–37%, Samcheon, Seoul, Republic of Korea) and aqueous ammonia (NH4OH, 25~30%, Duksan, Seoul, Republic of Korea) were employed for pH control. Absolute ethyl alcohol (99.9%, DAEJUNG, Siheung-si, Republic of Korea) was utilized to remove the surfactant. Tetraethylene pentamine (TEPA, 93%, Kanto Chemical, Tokyo, Japan) was used for adding amine functional groups to the synthesized silica nanomaterials.

### 3.2. Mesoporous Silica Synthesis Using a Calcination Process (Named MS-C)

The synthesis of mesoporous silica via a calcination process was performed as follows. A mixed solution of 40 mL of sodium silicate solution and 700 mL of distilled water was stirred at room temperature for half an hour. Then, 20 mL of CTACl was added and stirred at room temperature for 24 h. After stirring, the final solution was centrifuged at 10,000 rpm for 5 min to obtain a white slurry. The slurry was then dried in a convection oven at 80 °C for 8 h. The dried slurry was finely ground in a mortar and calcined at 600 °C for 5 h to remove surfactants. Finally, mesoporous silica nanomaterials were obtained. For more detailed information, please see reference [28].

### 3.3. Mesoporous Hollow Silica Synthesis Using a Calcination Process (Named MHS-C)

The synthesis via a calcination process for mesoporous hollow silica is as follows. Aqueous ammonia was added to a mixture of 500 mL of distilled water and 300 mL of ethanol to maintain pH 10. Then, 5.06 mL of CTACl was added dropwise to the mixture followed by stirring for half an hour. After adding 10 mL of TEOS into the mixture, the mixture was stirred at 35 °C for 10 h. The final product was collected by a decompression filter of the mixture and dried in a convection oven at 80 °C for 12 h. The dried product was finely ground and calcinated at 600 °C for 5 h to obtain mesoporous hollow silica materials.

### 3.4. Mesoporous Hollow Silica Synthesis through a Room Temperature Process (Named MHS-R)

The mesoporous hollow silica synthesized via a room-temperature surfactant removal process was carried out as follows. The overall process was identical to that of the MHS-C preparation except for the surfactant removal process. From making a mixture solution of distilled water, ethanol, aqueous ammonia, CTACl, and TEOS to drying in the convection oven, the same process as for MHS-C was performed. The dried product was then dispersed in 1 L of distilled water and stirred at 80 °C for 10 h. The product dispersed in water was gathered again through a decompression filter and dried in a convection oven at 100 °C for 12 h. The dried material was dispersed in a 1.0 M hydrochloric acid/ethanol solution and stirred at room temperature for 24 h to remove the surfactant. The final product was obtained by passing through a decompression filter and was then dried at room temperature. The product was washed twice with 1/1 distilled water/ethanol solution and dried at 80 °C for 12 h to obtain mesoporous hollow silica nanomaterials.

### 3.5. Synthesis to Introduce Amine Group

Amine groups were introduced into four types of silica nanomaterials, MS-C, MS-R, MHS-C, and MHS-R, with the same process. Each 0.5 g of silica nanomaterial was dispersed in ethanol (50 mL) and stirred at room temperature for 30 min. Then, 0.75 g of TEPA was added to the solution and stirred again for 1 h. The solvent of the mixed solution was removed using a rotary evaporator at 50 °C for 30 min. The remaining product was dried in a convection oven at 100 °C for 1 h to obtain silica nanomaterial into which an amine group was introduced.

### 3.6. Characterization

**Figure 8:**
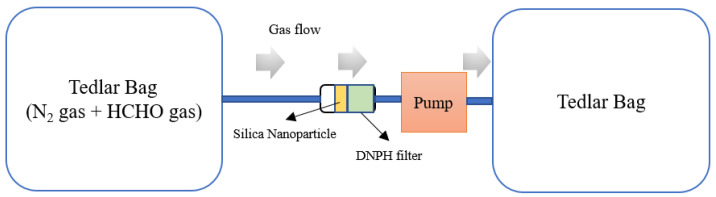
Schematic figure of HPLC setting for analyzing formaldehyde adsorption.

Pore and hollow (Pore/hollow) structures of mesoporous silica and mesoporous hollow silica were analyzed using a field emission transmission electron microscope (FE-TEM; HF-3300) at 300 kV as an operating voltage. The shells’ characteristics and thickness were also checked by using FE-TEM. The N_2_-adsorption-desorption isotherm (N_2_-sorption; QUANTACHROME, Qudrasorb SI) approach was utilized to verify specific surface areas and pore/hollow distributions of the mesoporous silica and mesoporous hollow silicas. The measured temperature was sustained at 77 K using liquid nitrogen and the adsorbed nitrogen was normalized to standard temperature and pressure. Before the analysis, an annealing process was performed at 200 °C for 6 h to get rid of moisture and impurities adsorbed on the sample surface. The Brunauer–Emmett–Teller (BET) specific surface area was deduced from the linear part (P/P_0_ = (0.05–0.30) of the BET equation. The volume and size of pores were determined from the Barrett–Joyner–Halenda (BJH) equation. An X-ray diffractometer (XRD; PANalytical X’pert PRO MRD) was employed to investigate the pore/hollow structure of the mesoporous silica and mesoporous hollow silicas. X-ray scans were carried out in 2θ scan mode with Cu-Kα rays (λ = 0.0154 nm). A Fourier transform infrared spectrometer (FT-IR; thermo, Nicolet iS50) was employed to confirm whether the mesoporous silica and mesoporous hollow silica were free of the surfactant and whether the amine functional groups were successfully introduced. Formaldehyde adsorption characteristics were analyzed using HPLC (Waters HPLC e2695, 2998 PDA detector). A mixture of nitrogen and formaldehyde containing formaldehyde at a concentration of 100 ppm was passed from the left Tedlar bag through 0.5 g of mesoporous silica with amine functional groups or mesoporous hollow silicas with amine functional groups at a rate of 500 mL per minute at 25 °C. Unadsorbed formaldehyde was derivatized by binding to 2,4-dinitrophenylhydrazine (DNPH) in the DNPH filter and analyzed by HPLC. To analyze the amount of remaining formaldehyde, a calibration curve was measured: Formaldehyde derivatization was generated using formaldehyde gas and DNPH, and it was dissolved in acetonitrile to draw a calibration curve. The HPLC was analyzed using a C18 column (component) with dimensions of 100 mm (length) and 4.6 mm (diameter), and measured by flow at a rate of 0.4 mL/min. A schematic diagram for the formaldehyde adsorption experiment is given in Figure 8.

## 4. Conclusions

In this work, mesoporous hollow silica materials having a remarkable specific surface area were synthesized using a surfactant and a silica precursor without a calcination process. For the synthesis of the mesoporous hollow silica material, a self-assembly method of surfactant was applied. The surfactant was then easily removed via HCl/EtOH solution extraction. Compared to MHS-C or MS-C synthesized by a calcination process, MHS-R showed an excellent adsorption performance of formaldehyde. The hollow structure and numerous amine functional groups attached to a superior specific surface area led to an outstanding performance for the adsorption of gaseous formaldehyde. Our results indicated that mesoporous hollow silica materials have great potential as a support to adsorb hazardous gases in the air. Additionally, the manufacturing cost of mesoporous hollow silica nanomaterials synthesized by the facile process is much lower than that of mesoporous hollow silica synthesized from core-shell structure [30]. This confirms the potential of such materials for application in various areas. Thus, we are planning to expand the study of applications of mesoporous hollow silica materials from hazardous gas adsorption to drug delivery.

## Figures and Tables

**Table 1:** Textural properties of mesoporous silica and mesoporous hollow silica.

	BET (m^2^/g)	Pore Size (nm)	Pore Volume (cc/g)
MS-C	1036.154	3.408	0.342
MHS-C	841.884	4.313	0.379
MHS-R	1223.950	3.835	0.363

## Data Availability

Not applicable.
